# AFM and Multiple Transmission-Reflection Infrared Spectroscopy (MTR-IR) Studies on Formation of Air-Stable Supported Lipid Bilayers

**DOI:** 10.3390/ijms10031407

**Published:** 2009-03-26

**Authors:** Peng-Feng Guo, Wen-Yi Huang, Hong-Bo Liu, Shou-Jun Xiao

**Affiliations:** State Key Laboratory of Coordination Chemistry, School of Chemistry and Chemical Engineering, Nanjing University, Nanjing 210093, P.R. China

**Keywords:** Supported lipid bilayers, Self-assembly, Atomic force microscopy, MTR-IR

## Abstract

Supported lipid bilayers (SLBs) were prepared by deposition of unilamellar vesicles on a silicon substrate. Atomic force microscopy (AFM) and a new Multiple Transmission-Reflection Infrared Spectroscopy (MTR-IR) developed by us were used to trace the dynamic formation of lipid bilayers on the silicon surfaces. The evolution from deformation of vesicles to formation of bilayers can be distinguished clearly by AFM imaging. MTR-IR provided high quality infrared spectra of ultrathin lipid bilayers with high sensitivity and high signal to noise ratio (SNR). The structural and orientational changes during vesicle’s fusion were monitored with MTR-IR. MTR-IR shows superiority over other infrared approaches for ultrathin films on standard silicon wafers in view of its economy and high sensitivity. Both MTR-IR and AFM results were consistent with each other and they provided more information for understanding the self-assembling procedure of SLBs.

## 1. Introduction

In biologically relevant media, the understanding of cell membranes at a molecular level has been complicated by the difficulty of applying surface spectroscopy techniques to fragile tissue samples. The use of cell-membrane mimics, such as unilamellar vesicles [1,2] and supported lipid bilayers (SLBs) [3–6], for fundamental studies of membrane-mediated biomolecular interactions has recently gained significant attention. With the emergence of various surface analytical tools, such as surface plasmon resonance, quartz crystal microbalance, impedance spectroscopy, atomic force microscopy, etc., planar SLBs have been shown to be an attractive complement for membrane mimics. The formation of SLBs has been greatly investigated, not only because of SLBs’ mimics to the cell membrane, but also owing to their potential in biotechnological applications such as biosensors [7–9], bio-MEMS [10–12], and immunoassays [13,14].

SLBs are planar, two-dimensional and extended bilayers of the same composition as vesicles, adsorbed on a suitable solid surface, such as glass [15], mica [16], self-assembled monolayer [17], polymer [18], quartz [19], and silicon dioxide [20,21]. In contrast, silicon-based SLBs are more attractive due to the extensive development of microelectronics and the potential application as biosensors. The two most common methods to form SLBs on hydrophilic surfaces are either Langmuir-Blodgett (LB) transfer technique [22,23] or vesicle fusion [24]. The LB transfer technique has been extensively investigated to be ideal for preparing membrane mimics. However, the latter technique, vesicle fusion, is more favorable to many researchers due to its simplicity in a lab without facilities and the flexible approaches to incorporate functional biomolecules [25]. The fundamental processes of forming SLBs through vesicle’s fusion are usually described in five steps: (I) preparation of unilamellar vesicles; (II) adsorption of unilamellar vesicles on the solid surface; (III) fusion between intact vesicles to form larger vesicles; (IV) deformation and rupture of larger vesicles to form bilayer patches on the support; (V) coalescence of bilayer patches to form a continuous bilayer [26].

A deep understanding of the dynamic formation of SLBs on silicon substrates should provide clues to prepare perfect membranes, which are fundamental for SLB applications. However, the lipid self-assembly process is governed by a number of key parameters such as vesicle size, lipid chemistry and charge, temperature, and vesicle’s concentration in the buffer solution. To monitor the dynamic formation of SLBs on silicon substrates, we applied atomic force microscopy (AFM) and our newly developed multiple transmission-reflection infrared spectroscopy (MTR-IR) to the study of the self-assembly process of egg yolk phosphatidylcholine (egg PC).

Atomic force microscope (AFM) is a direct imaging tool for visualizing lipid bilayers on a substrate. Under certain conditions AFM can follow the dynamic self-assembly process of SLB by sequential imaging [27]. On the other hand, lipid structures such as surface-bound vesicles or bilayer patches are known to be easily modified by the complex mutual interactions between vesicles, SLB patches, and the AFM tip. Tip-induced imaging artifacts will become very severe for the lasting and low-speed scan [20,28,29]. Imaging in tapping mode was reported to induce fewer disturbances such as occasionally tip-induced vesicle rupture in respect to imaging in contact mode and further modification of the AFM tip with poly(l-lysine)-graft-poly(ethylene glycol) (PLL-g-PEG) resulted in even better control in imaging lipid structures [30]. However AFM imaging only provides the topo-morphologies of SLBs, the molecular structural information is still lacking. MTR-IR is a complementary tool to obtain structural information such as bond types, side products, orientations of different groups, etc. [31,32]. In this method, the infrared-transparent sample (in our case it is the SLBs-coated silicon wafer) is inserted between two parallel gold mirrors. When infrared light reaches the silicon surface, it will be split into transmitted and reflected parts leading to multiple transmissions and reflections and finally guided by two gold mirrors to a detector. The set-up has a crucial parameter, simplified transmission times (*N*), which is defined as the times that the incoming light passes through the silicon wafer until it leaves for detector by assuming no reflection on silicon [31]. Obviously, N determines the signal intensity because N represents the interaction times of infrared light with molecules. It is a very sensitive infrared method for investigation of ultrathin films of soft materials coated on infrared-transparent semiconductors such as silicon or germanium. More details about MTR-IR can be found in our recently published work [31]. We used the polarized MTR-IR to trace the whole procedure of vesicle adsorption and fusion to form a continuous bilayer on silicon surfaces. High quality spectra of both *p-* and *s-*polarizations for the same SLB were achieved for analyses of molecular orientation. The dynamic assembly process of lipids from vesicles to continuous planar bilayers with the orientation evolution of methane and methylene vibration modes was followed by the dichroic ratio (A_s_/A_p_) in the egg PC bilayer. Both AFM and MTR-IR data are consistent with each other.

## 2. Results and Discussion

### 2.1. AFM Characterization

**Figure 1.**
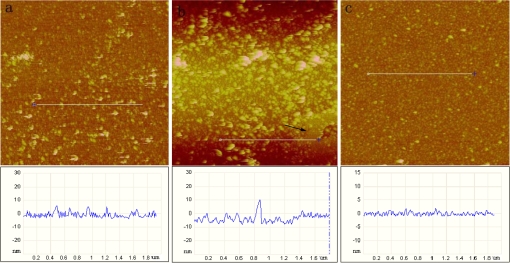
AFM height images (image size 3 × 3 μm^2^) of small unilamellar vesicles (SUVs, filtered by 100 nm polycarbonate membrane) and their corresponding section analyses after incubation of a silicon wafer in a vesicle solution (0.2 mg/mL) for (a) 10 min, (b) 30 min, and (c) 60 min respectively.

Figure 1 shows a typical self-assembly process for formation of a lipid bilayer by small unilamellar vesicles (SUVs). The silicon substrates were incubated for 10, 30, and 60 min respectively in a 0.2 mg/mL SUV solution (filtered by 100 nm polycarbonate membrane). Incubation for 10 min led to a complete coverage of SUVs on the wafer surface (Figure 1a). The cross section identified by the white line (measured from height images) shows the height of a flattened region of the liposome as 7 nm. After 30 min’s incubation (Figure 1b), the surface became much rougher because the small unilamellar vesicles were fused into larger vesicles. And to form a continuous planar lipid bilayer, the larger vesicles would collapse to form lipid bilayers (indicated by an arrow). Its correspondent cross section profile presents a larger vesicle and also vesicles’ rupturing to form bilayer patches. Figure 1c shows a continuous intact lipid bilayer after 60 min’s incubation. From these AFM images, we could draw a hypothesis for the formation of SLBs: first the small unilamellar vesicles were adsorbed intact onto the substrate, and then they fused together into bigger vesicles and finally the bigger vesicles ruptured to a planar continuous bilayer. The similar result was implied by previous reports [33].

**Figure 2.**
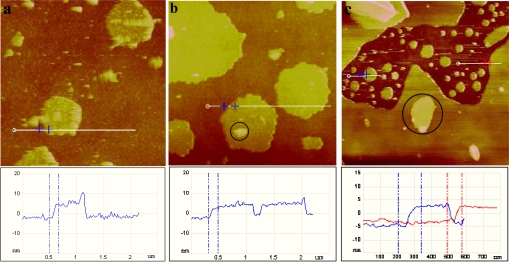

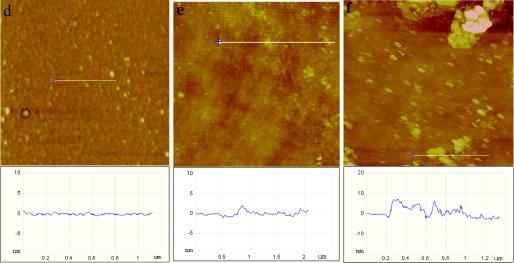
A series of AFM height images (image size 3 × 3 μm^2^) of bigger unilamellar vesicles adsorbed on silicon substrate and their corresponding cross-section analyses. Images were obtained after incubation of the substrate in a vesicle solution (0.2 mg/mL) for (a) 10 min, (b) 20 min, (c) 30 min, (d) 60 min, (e) 2 h, and (f) 5 h respectively.

We also attempted to use bigger unilamellar vesicles to form SLBs on the silicon substrate, where more characteristic information about the vesicle fusion and assembly would be observed. In our case, the prepared giant unilamellar vesicles (GUVs) were diluted and shaken to obtain unilamellar vesicles. Its dynamic assembly process is shown in Figure 2. When these unilamellar vesicles were adsorbed on a silicon wafer, besides the vesicles’ fusion, it seemed that vesicles were directly deformed and ruptured to form planar bilayers. Incubation for 10 min (Figure 2a) indicated the deformation of these vesicles and the formation of circular patches with a height of ~ 6.8 nm, a little bit higher than the 5.0 nm thickness of an ideal lipid bilayer. When the assembly time is prolonged to 20 min (Figure 2b), due to the fusion of adjacent vesicles or bilayer patches, the bilayer area became bigger and the height of 5.0 nm indicated they were extended flat bilayers. After incubation for 30 min, we found two kinds of bilayers, continuous and isolated ones, where their heights were identified by red line at ~ 5 nm and blue line at ~ 7 nm respectively. Bilayer patches coalesced with increasing vesicle’s exposure time (Figure 2a, b, c). Finally after 60 min a continuous planar film was formed in Figure 2d and it entirely covered the substrate surface.

Some multilayers and vesicles (circled in Figures 2b,c,d) were also observed. The number of multilayers and vesicles could be reduced by more rinsing, but they did not disappear completely. Longer exposure time led to more adsorption of vesicles and resulted in multilayer domains in Figures 2e and f at 2 h and 5 h, respectively. They are much rougher than the lipid bilayer, probably due to the random stacking of lipids.

### 2.2. Infrared spectroscopy analysis

**Figure 3.**
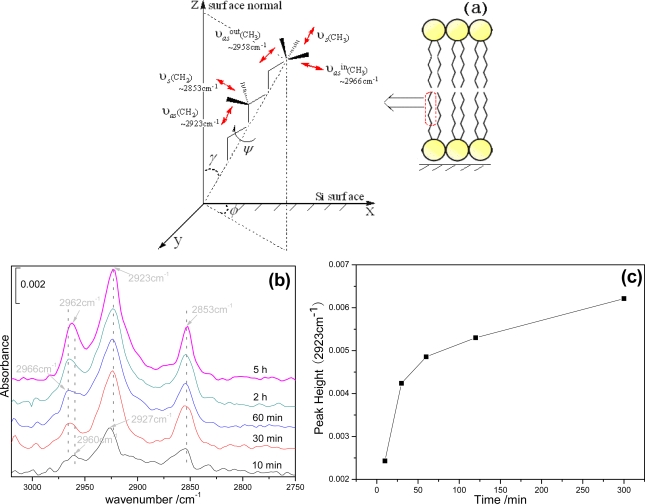
(a) The stretching vibration modes of the egg PC’s alkyl chain in 3D coordinate. (b) *P-*polarized MTR-IR spectra in the *C-H* stretching region after 10, 30, 60 min, 2 and 5 h’s incubation respectively. (c) Self-assembly procedure presented with the peak height at 2923 cm^−1^ against the assembly time.

MTR-IR spectra at *p-*polarization were used to trace the assembly process of egg PC SLBs on a silicon wafer (Figures 3b,c). Here we used *N =* 10 to perform the MTR-IR measurement. The *C-H* stretching modes from the non-polar hydrophobic hydrocarbon chains and the intermediate glycerol backbone of egg PC were analyzed. Three absorption bands are obvious between 2,800 and 3,000 cm^−1^, which are attributed to υ*_as_* (*CH*_3_), υ*_as_* (*CH*_2_), and υ*_s_* (*CH*_2_), respectively.

In Figure 3a, tilt angle (*γ*), azimuth angle (*φ*) and twist angle (*Ψ*) were defined to characterize the orientation of a hydrocarbon chain and the stretching vibrations of the methylene and methyl groups in a 3D coordinate. On the silicon surface, both normal (*z*) and parallel (*x*) components of transition dipole moments contribute to the absorbance at *p-*polarization, while only the parallel (*y*) components contribute to that at *s-*polarization. By assuming an extended all-trans conformation of the alkyl chain, the transition dipole moments of the methylene stretching modes are approximately perpendicular to the alkyl chain. While for CH_3_, it is much more complicated. According to the literature [34,35], the visible −*CH_3_* asymmetric stretching mode near 2,960 cm^−1^ can be split into two peaks, a higher wavenumber near 2,965 cm^−1^ for the in-plane stretching mode (υ*_as_^in^* (*CH*_3_)) and a lower wavenumber near 2,957 cm^−1^ for the out-of-plane stretching mode (υ*_as_^out^* (*CH*_3_)), respectively. υ*_as_^in^* (*CH*_3_) will have no projection in *y* axis, i.e., it will not appear in the *s*-polarized spectra for a very well-organized structure. In the *p*-polarized spectra of Figure 3b, the position of υ*_as_* (*CH*_3_) is slightly dependent on the assembly time. In the case of 10 min, the maximum −*CH_3_* asymmetric stretching peak emerges at 2,960 cm^−1^. As time goes by, it ultimately shifts to 2,966 cm^−1^ at 60 min, and finally comes back slightly to a lower wavenumber (2,962 cm^−1^) with further assembly. The slight shift of υ*_as_^in^* (*CH*_3_) indicates the alkyl chain’s organization from less ordered to slightly ordered and finally to less ordered again, corresponding to AFM’s observations in Figure 2.

The methylene stretching also provides molecular information such as conformation, orientation, and physical state of the long hydrocarbon chains. Usually, the head part of the egg PC molecule contains only four isolated methylene groups. Compared to the long alkyl methylene chain containing 14 CH_2_ units, their contribution to the IR spectrum is negligible [36]. It is well accepted that the lower −*CH_2_* stretching wavenumbers (2,917 and 2,850 cm^−1^) indicate highly ordered all-trans conformations of the alkyl chain, and the higher ones (2,928 and 2,856 cm^−1^) indicate the presence of gauche conformations and even more the melting of alkyl chains [37–39]. In Figure 3b, all −*CH_2_* stretching bands show higher wavenumbers, indicating disordered structures of −*CH_2_* in the whole assembly process. After 10 min’s assembly, the −*CH_2_* asymmetric stretching was located at 2,927 cm^−1^, which indicated the original vesicles adsorbed on silicon surfaces with random orientations. With increasing the assembly time, the position of υ*_as_* (*CH_2_*) was fixed at 2,923 cm^−1^ and υ*_s_*(*CH*_2_) at 2,853 cm^−1^, which indicated a physical state of alkyl chains between all-trans and gauche conformations. The transition temperature of egg PC is ~ 258 K. Under our room temperature (298K) condition, egg PC molecules should be in a liquid-like state and apparently some molecules will take the gauche conformations. This should be the main reason that the alkyl chains were less ordered. As seen in Figure 3c, the peak height of the *C-H* stretching mode is strongly dependent on the assembly time. The adsorption speed of vesicles at the initial stage was quick, and then became slower with prolonging the assembly time. And after about an hour, the absorbance reached saturation, meaning the formation of a continuous lipid bilayer. Although a slight increase was still observed, a combination analysis with AFM images in Figure 2 concluded that the blue shift was caused by random docking of vesicles on SLBs. Our MTR-IR results are in accordance with previous reports that the formation of continuous multilayers on the SLBs could not be achieved for most of lipid systems.

**Figure 4.**
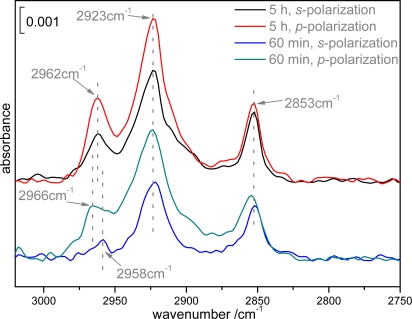
MTR-IR spectra at *s-* and *p-*polarizations respectively after incubation of silicon substrates in unilamellar solutions for 60 min (lower two traces) and 5 h (upper two traces).

In Figure 4, we compared both *s-* and *p-*polarized spectra at 1 and 5 h’s assembly. At 1 h, due to the ordered alkyl chain, υ*_as_^in^* (*CH*_3_) was suppressed in *s*-polarization. While at 5 h, υ*_as_^in^* (*CH*_3_) appeared in the *s-*polarized spectrum, indicating the disordered structure coming back again. By combined analysis of Figures 3a,b,c, 4, and 2, we inferred that, from the beginning to 60 min, unilamellar vesicles gradually aggregated on the surface, and at around 60 min, all alkyl chains of egg PC molecules were organized well to form a continuous lipid bilayer. With further assembly, some vesicles were randomly adsorbed onto the bilayer surface. And at this stage, majority of the adsorbed vesicles couldn’t fuse together and assemble into a second continuous bilayer, they just sat on the SLB surface and kept their shapes as isolated domains.

**Figure 5.**
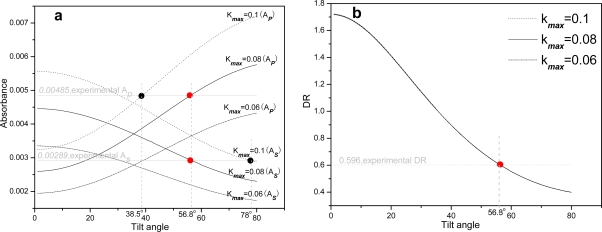
The simulated *A*_(_*_s_*_)_, *A*_(_*_p_*_)_ (a) and DR (b) against the tilt angle of egg PC SLBs according to our previous method [31]. The experimental data were labeled as dots to obtain the right tilt angle of 56.8º. The parameters used for calculations are: υ*_as_* (*CH*_2_) = 2923 cm^−1^, 70º incidence, thickness of the bilayer = 5 nm, *N =* 10, n_SLBs_ = 1.5, n_si_ = 3.42, n_air_ = 1.0, and n_Au_ = 2.018 + 21.087i.

We have established the MTR-IR method to deduce the molecular orientation on silicon surfaces [31]. Usually the intensities of υ*_as_*(*CH*_2_) and υ*_s_*(*CH*_2_) modes can be used to calculate the alkyl chain’s orientation. Ignoring the contribution of the glycerol backbone to methylene groups, we used the *s-*/*p-*polarized experimental and theoretical absorbance data to analyze the alkyl chain orientation. Calculated *A*_(_*_s_*_)_ and *A_(p)_* were rooted from the −*CH_2_* absorbance at 2,923 cm^−1^ (*A_(s)_* is the absorbance at *s-*polarization and *A_(p)_* the absorbance at *p-*polarization). The lipid bilayer was regarded as having uniaxial symmetry, and the alkyl chains’ orientation was associated with the absorption index *k* from the imaginary part of the complex refractive index (n̂ ***=*** *n* ***+*** *ik*). The maximum absorption index *k_max_*, when all transition moments are oriented along the same direction, can be obtained by fitting the experimental *s-* and *p-*polarized IR absorbance. The similar “spectrum fitting” method was also used by Allara and Ren [40,41]. The theoretical calculation was to find the correct *k_max_* to fit the experimental data. In Figure 5, the simulated curves with the absorbance of 2,923 cm^−1^ at both *s-* and *p-*polarizations *vs* the tilt angle were obtained by using different *k_max_* values. An incorrect *k_max_* will lead to different tilt angles when fitting the experimental data at *s-* and *p-*polarizations respectively. For example, when *k_max_* *=* 0.1, the tilt angle at *s-*polarization (corresponding to *A_(s)_*) is 78º, but at *p-*polarization (corresponding to *A_(p)_*) is 38.5º. When a value of *k_max_* *=* 0.08 was used, a consistent tilt angle of the alkyl chain was obtained as 56.8° both at *s-* and *p-*polarizations (*A_(s)_* is ~ 0.00289 and *A_(p)_* ~ 0.00485). This value is close to 54.5° which was reported for a DMPC bilayer [42]. Due to the presence of a large number of gauche conformations above the melting point of egg PC molecules, the tilt of hydrocarbon chains cannot be provided quantitatively. The tilt angle of 56.8º indicated a liquid-like state of hydrocarbon chains. A “dichroic ratio (DR) fitting” method in Figure 5b can also be used to get the tilt angle [31,32,35]. However the influence of *k_max_* on DR is very little. Within a *k_max_* range from 0.06 to 0.1 in Figure 5b, the resulting tilt angle of the alkyl chain has an error of only ± 0.1º.

## 3. Experimental Section

Egg yolk phosphatidylcholine (egg PC) was purchased from the Sinopharm Chemical Reagent Co., LtdS (Shanghai, P.R. China). A gentle hydration method was used to obtain unilamellar vesicles [43,44]. Briefly, egg PC was dissolved in chloroform, followed by evaporation of the solvent with a rotary evaporator to form a lipid film on the wall of a round bottom flask, then the dry film was rehydrated overnight in phosphate buffer saline (PBS, 10 mmol/L, pH 7.4, 100 mmol/L NaCl) at 25 °C to make a 1 mg/mL egg PC solution. During rehydration the lipid film was gradually stripped off from the wall of glass bottle layer by layer and formed cloud-like floaters in the solution. The resulted liposome called as giant unilamellar vesicles were transferred to a prepared test tube for more use. Small unilamellar vesicles (SUVs) were prepared by mechanically extruding the lipid suspensions through a 100 nm polycarbonate membrane (Whatman Inc., Kent, UK) 11 times.

Surface polished < 100 > silicon wafer (*p-*type, B doped, 7 ~ 13 Ω cm resistivity, and 0.5 mm thickness) was cut into 1 × 1 cm^2^ for AFM measurement and 2 × 3 cm^2^ for MTR-IR analysis. The chips were then cleaned with heated “piranha solution” [1:3 (v/v) mixture of 30 % H_2_O_2_ and 95 % H_2_SO_4_] for 30 min to remove impurities and to obtain hydrophilic silicon dioxide surfaces. Before SLB’s assembly, unilamellar vesicles were diluted to 0.2 mmol/L, and then the freshly cleaned silicon substrate was incubated in the solution at room temperature (25 °C). Before the use of GUVs, the solution was diluted and shaken five minutes by hand to form smaller vesicles. After assembly, immediately the sample was rinsed thoroughly with PBS, subsequently dipped into de-ionized water 30 s for a slight desalting, and then dried under a stream of nitrogen. The de-ionized water used in all experiments is the MilliQ water (≥ 18.2 MΩ cm).

The AFM experiments were carried out on a Multimode V (Veeco Instruments Ins.) in tapping mode. The forces were adjusted during each scan by manually minimizing the set point. Typical scan rates of 1 ~ 2 Hz at a resolution of 256 – 512 pixels/line were used. All images shown are subjected to a first order plane-fitting procedure to compensate for the sample tilt.

The infrared spectra were recorded on a Bruker IFS66/S instrument at 4 cm^−1^ resolution. The DTGS detector was used for all experiments. Self-designed multiple transmission-reflection IR accessory was built up to accomplish the enhancement of absorption signals [31]. A scanning time of 100 scans was used to get spectra with high signal to noise ratios. The incidence angle of the IR beam was fixed at 70º, a clean silicon wafer as a reference, and a KRS-5 grid polarizer to polarize the incidence light.

## 4. Conclusions

In summary, egg PC SLBs were prepared on a silicon substrate by vesicle fusion. The kinetic self-assembly processes of the vesicles’ adsorption, fusion, and rupturing to form bilayers were characterized by AFM. In the meantime, our newly developed infrared method, MTR-IR, was first used to trace the SLBs’ formation. MTR-IR spectra are powerful to detect the adsorbed phosphatidylcholine molecules and to analyze the long alkyl chain’s orientation in respect to the silicon substrate surface. Consistent results about SLBs’ assembly were obtained by AFM and MTR-IR respectively. The infrared details obtained by MTR-IR are useful to understand and to control SLBs’ formation.
